# Supplementary material for: Incidence of long-term post-acute sequelae of SARS-CoV-2 infection related to pain and other symptoms: A systematic review and meta-analysis
Source: PLoS One. 2023 Nov 29;18(11):e0250909. doi: 10.1371/journal.pone.0250909 (PMC10686440; doi:10.1371/journal.pone.0250909)
Supplement: S1 Appendix — (DOCX) [file pone.0250909.s001.docx]

**S1A Appendix. Literature Search Strategy**

1. **PubMed**

“long COVID”[All Fields] OR “long COVID-19”[All Fields] OR “long-haul covid”[All Fields] OR “Chronic COVID syndrome”[All Fields] OR “Post-COVID-19 syndrome”[All Fields] OR (“Long-term complications”[All Fields] AND “COVID”[All Fields]) OR (“long-term consequences”[All Fields] AND ”COVID”[All Fields]) OR (“long-term sequelae”[All Fields] AND “COVID”[All Fields])

1. **EMBASE**

(‘long covid’ OR ‘long covid -19’ OR ‘long-haul covid’ OR ‘Chronic covid syndrome’ OR ‘Post- covid -19 syndrome’ OR (‘Long-term complications’AND ‘covid’) OR (‘long-term consequences’ AND ‘covid’) OR (‘long-term sequelae’ AND ‘covid’)

1. **Scopus**

( "long COVID"  OR  "long COVID-19"  OR  "long-haul covid"  OR  "Chronic COVID syndrome"  OR  "Post-COVID-19 syndrome"  OR  ( "Long-term complications"  AND  "COVID" )  OR  ( "long-term consequences"  AND  "COVID" ) )

1. **CHINAL**

long COVID [TI] OR long COVID-19 [TI] OR long-haul covid [TI] OR Chronic COVID syndrome [TI] OR Post-COVID-19 syndrome [TI] OR (Long-term complications of COVID [TI]) OR (long-term consequences of COVID [TI]) OR (long-term sequelae of COVID [TI])

1. **MedRχiv and BioRχiv**

( "long COVID"  OR  "long COVID-19"  OR  "long-haul covid"  OR  "Chronic COVID syndrome"  OR  "Post-COVID-19 syndrome"  OR  ( "Long-term complications"  AND  "COVID" )  OR  ( "long-term consequences"  AND  "COVID" ) )

**S1B Appendix. R code**

library(meta)

# meta-analysis of proportions, inverse variance method, DerSimonian-Laird estimator for tau^2, Logit #transformation

meta<-metaprop(n, N, data=Abdominal_pain,studlab =paste(Author),method="Inverse",

comb.fixed = F,comb.random = T, method.tau="DL", sm="PLOGIT")

meta

forest(meta)

# Mixed-Effects model meta-regression for followup, Age and Sex as covariates

metareg1<-metareg(meta,followup)

metareg1

metareg2<-metareg(meta,meanAge)

metareg2

metareg3<-metareg(meta, maleGender)

metareg3

bubble1<-bubble(metareg1)

bubble2<-bubble(metareg2)

bubble3<-bubble(metareg3)

# Plot to assess funnel plot asymmetry

funnel(meta)

# Egger's linear regression test of funnel plot asymmetry

metabias(meta, method.bias = "linreg", k.min=3)

**S1C Appendix. The system of Newcastle-Ottawa quality assessment scale in cohort studies**

Note: A study can be awarded a maximum of one star for each numbered item within the Selection and

Outcome categories. A maximum of two stars (*) can be given for Comparability

**Selection**

1) Representativeness of the exposed cohort

a) truly representative of the average _______________ (describe) in the community *****

b) somewhat representative of the average ______________ in the community *****

c) selected group of users eg nurses, volunteers

d) no description of the derivation of the cohort

2) Selection of the non exposed cohort

a) drawn from the same community as the exposed cohort *****

b) drawn from a different source

c) no description of the derivation of the non exposed cohort

3) Ascertainment of exposure

a) secure record (eg surgical records) *****

b) structured interview *****

c) written self report

d) no description

4) Demonstration that outcome of interest was not present at start of study

a) yes *****

b) no

**Comparability**

1) Comparability of cohorts on the basis of the design or analysis

a) study controls for _____________ (select the most important factor) *****

b) study controls for any additional factor ***** (This criteria could be modified to indicate specific

control for a second important factor.)

**Outcome**

1) Assessment of outcome

a) independent blind assessment *****

b) record linkage *****

c) self report

d) no description

2) Was follow-up long enough for outcomes to occur

a) yes (select an adequate follow up period for outcome of interest) *****

b) no

3) Adequacy of follow up of cohorts

a) complete follow up - all subjects accounted for *****

b) subjects lost to follow up unlikely to introduce bias - small number lost - > 80 % (select an

adequate %) follow up, or description provided of those lost) *****

c) follow up rate < 80% (select an adequate %) and no description of those lost

d) no statement
