# Supplementary material for: Incidence of long-term post-acute sequelae of SARS-CoV-2 infection related to pain and other symptoms: A systematic review and meta-analysis
Source: PLoS One. 2023 Nov 29;18(11):e0250909. doi: 10.1371/journal.pone.0250909 (PMC10686440; doi:10.1371/journal.pone.0250909)
Supplement: S1 Table — (DOCX) [file pone.0250909.s002.docx]

**S1A Table. Summary of studies included in the meta-analysis**

| **First  author** | **Year published** | **Location** | **Patinet setting** | **Sample size  (n)** | **Diagnotic criteria of SARS-CoV-2** | **Respiratory  support** | **Age (mean or median)** | **Sex,  % male** | **Follow-up period, (maximum) (month)** | **Patient follow-up (n/total)** |  |  |  |  |  |  |  |  |  |
| --- | --- | --- | --- | --- | --- | --- | --- | --- | --- | --- | --- | --- | --- | --- | --- | --- | --- | --- | --- |
| Arnold, D^19^ | 2020 | UK | Non-hospitalized | 110 | Positive PCR and clinico-radiological diagnosis | Oxygen alone, CPAP or IV | 60 | 56 | 3 | 110/110 |  |  |  |  |  |  |  |  |  |
| Boscolo-Rizzo, P^20^ | 2020 | Italy | Hospitalized | 187 | PCR | NS | 56 | 44.9 | 1 | 187/202 |  |  |  |  |  |  |  |  |  |
| Carfi, A^21^ | 2020 | Italy | Hospitalized | 143 | RT-PCR | Oxygen alone, CPAP or IV | 56.5 | 62.9 | 2 | 143/143 |  |  |  |  |  |  |  |  |  |
| Carvalho-Schneider, C^22^ | 2021 | France | Hospitalized and non-hospitalized | 150 | RT-PCR | NS | 48.8 | 56 | 2 | 130/293 |  |  |  |  |  |  |  |  |  |
| Cheng, DO^23^ | 2020 | UK | Hospitalized | 109 | PCR and clinical symptoms | NS | 73 | 55.8 | 2.3 | 109/1946 |  |  |  |  |  |  |  |  |  |
| Chiesa-Estomb, CN^24^ | 2020 | Spain | Hospitalized and non-hospitalized | 751 | RT-PCR and serology | NS | 41 | 36.5 | 1.6 | 751/1231 |  |  |  |  |  |  |  |  |  |
| Chopra, V^54^ | 2020 | USA | Non-hospitalized | 488 | No description | NS | 62 | 51.8 | 2 | 488/1468 |  |  |  |  |  |  |  |  |  |
| Cirulli, E^25^ | 2020 | USA | Hospitalized and non-hospitalized | 357 | No description | NS | 56 | 35.9 | 3 | 216/357 |  |  |  |  |  |  |  |  |  |
| Davis, HE^26^ | 2020 | 56  countries | Hospitalized and non-hospitalized | 3762 | PCR, antigen, antibody positive | NS | 50.5 | 78.9 | 7 | 3762/3762 |  |  |  |  |  |  |  |  |  |
| Dennis, A^27^ | 2020 | UK | Hospitalized and non-hospitalized | 201 | RT-PCR, serology and symptoms | NS | 44 | 30.3 | 5.3 | 201/unreported |  |  |  |  |  |  |  |  |  |
| Eiros, R^28^ | 2020 | Spain | Hospitalized and non-hospitalized | 139 | RT-PCR and serology | Oxygen | 52 | 28.1 | 2.8 | 139/142 |  |  |  |  |  |  |  |  |  |
| Garrigues, E^55^ | 2020 | France | Hospitalized | 120 | RT-PCR and CT | Oxygen | 63.2 | 62.5 | 3.8 | 120/279 |  |  |  |  |  |  |  |  |  |
| Geortz, YNJ^29^ | 2020 | Netherlands | Hospitalized and non-hospitalized | 2113 | PCR and CT | NS | 47 | 14.7 | 3.2 | 2113/2113 |  |  |  |  |  |  |  |  |  |
| Halpin, SJ^30^ | 2020 | UK | Hospitalized | 100 | PCR positive | Oxygen alone, CPAP or IV | 66.7 | 54 | 2 | 100/191 |  |  |  |  |  |  |  |  |  |
| Huang, C^31^ | 2021 | China | Hospitalized | 1733 | SARS-CoV2 antibody | NS | 57 | 52 | 6.6 | 1733/2469 |  |  |  |  |  |  |  |  |  |
| Klein, H^32^ | 2020 | Israel | Hospitalized and non-hospitalized | 112 | RT-PCR positive | NS | 35 | 64.3 | 6 | 112/114 |  |  |  |  |  |  |  |  |  |
| Lavoto, A^33^ | 2020 | Italy | except for ICU | 121 | Swab PCR positive | NS | 46.7 | 40.5 | 1.4 | 121/121 |  |  |  |  |  |  |  |  |  |
| Mandal, S^34^ | 2020 | UK | Hospitalized | 384 | Swab PCR  positive | Oxygen alone, CPAP or IV | 59.9 | 62 | 1.5 | 384/479 |  |  |  |  |  |  |  |  |  |
| Moreno-Pérez, O^56^ | 2021 | Spain | Hospitalized | 141 | PCR or subsequent seroconversion | NS | 56 | 52.5 | 4.5 | 141/277 |  |  |  |  |  |  |  |  |  |
| Moradian, ST^35^ | 2021 | Iran | Hospitalized | 200 | RT-PCR | NS | 55.6 | 80 | 1.5 | 200/300 |  |  |  |  |  |  |  |  |  |
| Neto, DB^36^ | 2020 | Brazil | Hospitalized | 545 | RT-PCR | NS | 37.7 | 36.3 | 4 | 545/669 |  |  |  |  |  |  |  |  |  |
| Petersen, M^37^ | 2020 | Denmark | Non-hospitalized | 180 | RT-PCR of an oropharyngeal swab | NS | 39.9 | 45.6 | 7 | 180/180 |  |  |  |  |  |  |  |  |  |
| Pilotto, A^38^ | 2020 | Italy | Hospitalized | 165 | NS | Oxygen alone, CPAP or IV | 64.8 | 24 | 6 | 165/208 |  |  |  |  |  |  |  |  |  |
| Poncet-Megemont, L^39^ | 2020 | France | Hospitalized and non-hospitalized | 139 | PCR and chest CT | NS | 48.5 | 37.4 | 1.1 | 139/180 |  |  |  |  |  |  |  |  |  |
| Rahmani, H^40^ | 2020 | Iran | Hospitalized | 173 | PCR , clinical data and chest CT | Oxygen alone, CPAP or IV | 60 | 53.1 | 1.9 | 173/213 |  |  |  |  |  |  |  |  |  |
| Savarraj, JPJ^41^ | 2020 | USA | Hospitalized | 48 | RT-PCR | NS | 50 | 34.3 | 3 | 48/140 |  |  |  |  |  |  |  |  |  |
| Salmon-Ceron, D^42^ | 2021 | France | Hospitalized and non-hospitalized | 70 | positive PCR and serology | Oxygen alone, CPAP or IV | 45 | 78.6 | 2 | 70/70 |  |  |  |  |  |  |  |  |  |
| Stavem, K^43^ | 2020 | Norway | Non-hospitalized | 451 | PCR | NS | 49.8 | 44 | 4.2 | 451/451 |  |  |  |  |  |  |  |  |  |
| Sudre, C^44^ | 2020 | UK, US, Sweden | NS | 4182 | PCR | NS | 42 | 28.5 | 2 | 4182/4182 |  |  |  |  |  |  |  |  |  |
| Tenforde, MW^45^ | 2020 | US | Non-hospitalized | 270 | RT-PCR positive | NS | 39.6 | 48.1 | 1 | 270/274 |  |  |  |  |  |  |  |  |  |
| Tomasoni, D^46^ | 2020 | Italy | Hospitalized | 105 | NS | Oxygen alone, CPAP or IV | 55 | 73.3 | 3 | 105/105 |  |  |  |  |  |  |  |  |  |
| Townsend, L^47^ | 2020 | Ireland | Hospitalized | 128 | RT-PCR | NS | 49.5 | 53.9 | 1.5 | 128/223 |  |  |  |  |  |  |  |  |  |
| Wang, X^48^ | 2020 | China | Hospitalized | 131 | NS | NS | 49 | 45 | 1 | 131/147 |  |  |  |  |  |  |  |  |  |
| Weerahandi, H^49^ | 2020 | USA | Hospitalized | 152 | laboratory- confirmed | Oxygen | 62 | 63 | 1.3 | 152/161 |  |  |  |  |  |  |  |  |  |
| Wu, C^50^ | 2020 | China | Hospitalized | 370 | RT-PCR | NS | 50.5 | 54.9 | 0.8 | 370/370 |  |  |  |  |  |  |  |  |  |
| Xiong, Q^51^ | 2020 | China | Hospitalized | 538 | according to WHO guidance | NS | 52 | 45.5 | 3.6 | 538/2641 |  |  |  |  |  |  |  |  |  |
| Yan, N^52^ | 2020 | China | Hospitalized | 337 | RT-PCR positive | NS | 44 | 45.7 | 0.5 | 296/337 |  |  |  |  |  |  |  |  |  |
| Zhao, Y^53^ | 2020 | China | Hospitalized | 55 | RT-PCR positive | Oxygen | 47.8 | 58.2 | 3 | 55/55 |  |  |  |  |  |  |  |  |  |
|  |  |  |  |  |  |  |  |  |  |  |  |  |  |  |  |  |  |  |  |
| RT-PCR: Reverse Transcription Polymerase Chain Reaction, CPAP: Continuous Positive Airway Pressure, IV: Invasive Ventilation, |  |  |  |  |  |  |  |  |  |  |  |  |  |  |  |  |  |  |  |
| NS:not specified. | | | | | | | | | | |  |  |  |  |  |  |  |  |  |
|  | |  |  |  |  |  |  |  |  |  |  |  |  |  |  |  |  |  |  |

**S1B Table. The Results of the Newcastle-Ottawa Quality Assessment Scale for Included Studies**

| **First  author** | **1) Representativeness** | **2) None exposed cohort** | **3) Ascertainment of exposure** | **4) Demonstration** | **5) Comparability** | **6) Assessment of outcome** | **7) Was follow-up long enough** | **8) Adequacy of follow-up** | **Score** |
| --- | --- | --- | --- | --- | --- | --- | --- | --- | --- |
| Arnold, D^19^ | a | NA | a | b | NA | a | a | a | 6 |
| Boscolo-Rizzo, P^20^ | a | NA | b | b | NA | c | a | b | 5 |
| Carfi, A^21^ | a | NA | a | a | NA | a | a | a | 6 |
| Carvalho-Schneider, C^22^ | a | NA | b | a | NA | a | a | a | 6 |
| Cheng, DO^23^ | a | NA | b | a | NA | a | a | a | 6 |
| Chiesa-Estomb, CN^24^ | a | NA | b | a | NA | a | a | b | 6 |
| Chopra, V^54^ | a | NA | a | b | NA | c | a | b | 5 |
| Cirulli, E^25^ | a | NA | b | b | NA | c | a | a | 5 |
| Davis, HE^26^ | b | NA | b | b | NA | c | a | a | 5 |
| Dennis, A^27^ | a | NA | a | a | NA | c | a | d | 3 |
| Eiros, R^28^ | c | NA | b | a | NA | b | a | b | 5 |
| Garrigues, E^55^ | a | NA | a | b | NA | c | a | b | 5 |
| Geortz, YNJ^29^ | b | NA | c | a | NA | c | a | d | 3 |
| Halpin, SJ^30^ | a | NA | b | a | NA | c | a | c | 4 |
| Huang, C^31^ | a | NA | a | a | NA | a | a | c | 5 |
| Klein, H^32^ | a | NA | b | a | NA | c | a | b | 5 |
| Lavoto, A^33^ | c | NA | a | a | NA | c | a | d | 3 |
| Mandal, S^34^ | a | NA | b | a | NA | c | a | b | 5 |
| Moreno-Pérez, O^56^ | a | NA | a | b | NA | b | a | b | 6 |
| Moradian, ST^35^ | a | NA | b | a | NA | a | a | a | 6 |
| Neto, DB^36^ | b | NA | c | b | NA | c | a | b | 4 |
| Petersen, M^37^ | a | NA | a | b | NA | c | a | a | 5 |
| Pilotto, A^38^ | a | NA | b | a | NA | a | a | a | 6 |
| Poncet-Megemont, L^39^ | a | NA | b | a | NA | c | a | b | 5 |
| Rahmani, H^40^ | b | NA | a | a | NA | a | a | b | 6 |
| Savarraj, JPJ^41^ | a | NA | b | a | NA | c | a | b | 5 |
| Salmon-Ceron, D^42^ | a | NA | a | a | NA | b | a | b | 6 |
| Stavem, K^43^ | a | NA | a | b | NA | c | a | a | 5 |
| Sudre, C^44^ | a | NA | b | b | NA | c | a | a | 5 |
| Tenforde, MW^45^ | b | NA | b | a | NA | c | a | b | 5 |
| Tomasoni, D^46^ | a | NA | b | b | NA | a | a | a | 6 |
| Townsend, L^47^ | a | NA | b | a | NA | a | a | a | 6 |
| Wang, X^48^ | a | NA | a | b | NA | a | a | b | 6 |
| Weerahandi, H^49^ | b | NA | b | a | NA | c | a | b | 5 |
| Wu, C^50^ | a | NA | b | a | NA | d | a | b | 6 |
| Xiong, Q^51^ | a | NA | b | a | NA | c | a | b | 5 |
| Yan, N^52^ | a | NA | b | a | NA | c | b | b | 5 |
| Zhao, Y^53^ | a | NA | a | a | NA | b | a | a | 6 |
|  |  |  |  |  |  |  |  |  |  |
| NA: not applicable. |  |  |  |  |  |  |  |  |  |

**S1C Table**. Most frequent symptoms ranked in each study

| **Author** | **Ranking** | | |
| --- | --- | --- | --- |
|  | **1** | **2** | **3** |
| Arnold, DT | Fatigue | Dyspnea | Insomnia |
| Boscolo-Rizzo, P | Anosmia | Agesusia | Cough |
| Carfi, A | Fatigue | Dyspnea | Arthalgia |
| Carvalho-Schneider, C | Flulike symptoms | Other respiratory signs | Anosmia |
| Cheng, DO | Fatigue | Dyspnea | Cough |
| Chopra, V | Dyspnea | Cough | Anosmia |
| Cirulli, ET | Cough | Dyspnea | Anosmia |
| Davis, HE | Fatigue | Post exertional malaise | Fever |
| Dennis, A | Fatigue | Myalgia | Dyspnea |
| Eiros, R | Fatigue | Anosmia | Ageusia |
| Garrigues, E | Fatigue | Dyspnea | Memory  loss |
| Geortz, YMJ | Fatigue | Dyspnea | Headache |
| Halpin, SJ | Fatigue | Dyspnea | PTSD |
| Huang, C | Fatigue | Insomnia | Hair loss |
| Klein, H | Fatigue | Anosmia | Dyspnea |
| Lovato, A | Fever | Fatigue | Cough |
| Mandal, S | Insomnia | Cough | Dyspnea |
| Moreno-Pérez, O | Fatigue | Anosmia | Ageusia |
| Moradian, ST | Dyspnea | Weakness | Cough |
| Neto, DB | Anosmia | Ageusia | Rhinorrhea |
| Petersen, MS | Fatigue | Anosmia | Ageusia |
| Pilotto, A | Fatigue | Memory  loss | Insomnia |
| Poncet-Megemont, L | Asthenia | Fever | Anosmia |
| Rahmani, H | Dyspnea | Cough | Fever |
| Savarraj, JPJ | Any neurologic symptom | Fatigue | PTSD |
| Salmon-Ceron, D | Asthenia | Sensory disturbance | Chest pain |
| Stavem, K | Dyspnea | Anosmia | Dysgeusia |
| Sudre, CH | Fatigue | Headache | Dyspnea |
| Tenforde, MW | Cough | Fatigue | Congestion |
| Tomasoni, D | Anosmia | Ageusia | Fever |
| Wang, X | Cough | Fatigue | Sputum |
| Wu, C | Cough | Dyspnea | Sputum |
| Xiong, Q | Fatigue | Sweating | Dyspnea |
| Yan, N | Cough | Congestion | Sputum |
| Zhao, Y | Fever | Cough | Weakness |
