# Supplementary material for: Incidence of long-term post-acute sequelae of SARS-CoV-2 infection related to pain and other symptoms: A systematic review and meta-analysis
Source: PLoS One. 2023 Nov 29;18(11):e0250909. doi: 10.1371/journal.pone.0250909 (PMC10686440; doi:10.1371/journal.pone.0250909)
Supplement: S1 Fig — (DOCX) [file pone.0250909.s003.docx]

**S1A Fig. Forest Plot on Abdominal Pain**

Point sizes are proportional to an inverse of the precision of the estimates and bar correspond to 95% confidence intervals.

**S1B Fig. Bubble Plots (follow-up period) on Abdominal Pain**

Point sizes are proportional to an inverse of the precision of the estimates.

The regression coefficient was 0.25 (95% confidence interval:-0.12-0.62).(*P*=0.18).

**S1C Fig. Bubble Plots (age) on Abdominal Pain**

Point sizes are proportional to an inverse of the precision of the estimates.

The regression coefficient was -0.08 (95% confidence interval:-0.19-0.04).(*P*=0.20).

**S1D Fig. Bubble Plots (sex) on Abdominal Pain**

Point sizes are proportional to an inverse of the precision of the estimates.

The regression coefficient was -0.01 (95% confidence interval:--0.06-0.03).(*P*=0.59).

**S1E Fig. Funnel Plot of studies reporting Abdominal Pain**

Egger’s *P* was 0.09, indicating the presence of publication bias.

**S2A. Forest Plot on Arthralgia**

Point sizes are proportional to an inverse of the precision of the estimates and bar correspond to 95% confidence intervals.

**S2B. Bubble Plots (follow-up period) on Arthralgia**

Point sizes are proportional to an inverse of the precision of the estimates.

The regression coefficient was 0.18 (95% confidence interval:-0.22-0.57).(*P*=0.37).

**S2C. Bubble Plots (age) on Arthralgia**

Point sizes are proportional to an inverse of the precision of the estimates.

The regression coefficient was -0.02 (95% confidence interval:-0.13-0.09).(*P*=0.78).

**S2D. Bubble Plots (sex) on Arthralgia**

Point sizes are proportional to an inverse of the precision of the estimates.

The regression coefficient was 0.003 (95% confidence interval:-0.05-0.05).(*P*=0.90).

**S2E. Funnel Plot of studies reporting on Arthralgia**

Egger’s *P* was 0.9, indicating the absence of publication bias.

**S3A. Forest Plot on Chest Pain**

Point sizes are proportional to an inverse of the precision of the estimates and bar correspond to 95% confidence intervals.

**S3B. Bubble Plots (follow-up period) on Chest Pain**

Point sizes are proportional to an inverse of the precision of the estimates.

The regression coefficient was 0.14 (95% confidence interval:-0.16-0.44).(*P*=0.36).

**S3C. Bubble Plots (age) on Chest Pain**

Point sizes are proportional to an inverse of the precision of the estimates.

The regression coefficient was -0.06 (95% confidence interval:-0.14-0.02).(*P*=0.12).

**S3D. Bubble Plots (sex) on Chest Pain**

Point sizes are proportional to an inverse of the precision of the estimates.

The regression coefficient was -0.001 (95% confidence interval:-0.03-0.03).(*P*=0.96).

**S3E. Funnel Plot of studies reporting on Chest Pain**

Egger’s *P* was 0.02, indicating the presence of publication bias.

**S4A. Forest Plot on Ear Pain**

Point sizes are proportional to an inverse of the precision of the estimates and bar correspond to 95% confidence intervals.

**S4B. Bubble Plots (follow-up period) on Ear Pain**

Point sizes are proportional to an inverse of the precision of the estimates.

The regression coefficient was 0.58 (95% confidence interval:-0.35-1.51).(*P*=0.22).

**S4C. Bubble Plots (age) on Ear Pain**

Point sizes are proportional to an inverse of the precision of the estimates.

The regression coefficient was -0.31 (95% confidence interval:-1.06-0.44).(*P*=0.42).

**S4D. Bubble Plots (sex) on Ear Pain**

Point sizes are proportional to an inverse of the precision of the estimates.

The regression coefficient was -0.003 (95% confidence interval:-0.090-0.08).(*P*=0.94).

**S4E. Funnel Plot of studies reporting on Ear Pain**

Egger’s *P* was 0.28, indicating the absence of publication bias.

**S5A. Forest Plot on Headache**

Point sizes are proportional to an inverse of the precision of the estimates and bar correspond to 95% confidence intervals.

**S5B. Bubble Plots (follow-up period) on Headache**

Point sizes are proportional to an inverse of the precision of the estimates.

The regression coefficient was 0.07 (95% confidence interval:-0.33-0.47).(*P*=0.74).

**S5C. Bubble Plots (age) on Headache**

Point sizes are proportional to an inverse of the precision of the estimates.

The regression coefficient was -0.05 (95% confidence interval:-0.14-0.03).(*P*=0.23).

**S5D. Bubble Plots (sex) on Headache**

Point sizes are proportional to an inverse of the precision of the estimates.

The regression coefficient was -0.01 (95% confidence interval:-0.05-0.03).(*P*=0.63).

**S5E. Funnel Plot of studies reporting on Headache**

Egger’s *P* was 0.0003, indicating the presence of publication bias.

**S6A. Forest Plot on Myalgia**

Point sizes are proportional to an inverse of the precision of the estimates and bar correspond to 95% confidence intervals.

**S6B. Bubble Plots (follow-up period) on Myalgia**

Point sizes are proportional to an inverse of the precision of the estimates.

The regression coefficient was 0.38 (95% confidence interval:--0.07-0.82).(*P*=0.09).

**S6C. Bubble Plots (age) on Myalgia**

Point sizes are proportional to an inverse of the precision of the estimates.

The regression coefficient was 0.03 (95% confidence interval:-0.09-0.14).(*P*=0.62).

**S6D. Bubble Plots (sex) on Myalgia**

Point sizes are proportional to an inverse of the precision of the estimates.

The regression coefficient was -0.01 (95% confidence interval:-0.07-0.05).(*P*=0.76).

**S6E. Funnel Plot of studies reporting on Myalgia**

Egger’s *P* was 0.008, indicating the presence of publication bias.

**S7A. Forest Plot on Neuralgia**

Point sizes are proportional to an inverse of the precision of the estimates and bar correspond to 95% confidence intervals.

**S7B. Bubble Plots (follow-up period) on Neuralgia**

Point sizes are proportional to an inverse of the precision of the estimates.

The regression coefficient was 0.39 (95% confidence interval:0.29-0.48).(*P*<0.001).

**S7C. Bubble Plots (age) on Neuralgia**

Point sizes are proportional to an inverse of the precision of the estimates.

The regression coefficient was -0.40 (95% confidence interval:-0.60—0.19).(*P*=0.0002).

**S7D. Bubble Plots (sex) on Neuralgia**

Point sizes are proportional to an inverse of the precision of the estimates.

The regression coefficient was 0.005 (95% confidence interval:0.001-0.10).(*P*=0.045).

**S7E. Funnel Plot of studies reporting on Neuralgia**

Egger’s *P* was 0.14, indicating the absence of publication bias.

**S8A. Forest Plot on Sore Throat**

Point sizes are proportional to an inverse of the precision of the estimates and bar correspond to 95% confidence intervals.

**S8B. Bubble Plots (follow-up period) on Sore Throat**

Point sizes are proportional to an inverse of the precision of the estimates.

The regression coefficient was 0.20 (95% confidence interval:-0.17-0.58).(*P*=0.29).

**S8C. Bubble Plots (age) on Sore Throat**

Point sizes are proportional to an inverse of the precision of the estimates.

The regression coefficient was -0.04 (95% confidence interval:-0.15-0.08).(*P*=0.53).

**S8D. Bubble Plots (sex) on Sore Throat**

Point sizes are proportional to an inverse of the precision of the estimates.

The regression coefficient was -0.02 (95% confidence interval: -0.05-0.02).(*P*=0.40).

**S8E. Funnel Plot of studies reporting on Sore Throat**

Egger’s *P* was 0.40, indicating the absence of publication bias.

**S9A. Forest Plot on Ageusia**

Point sizes are proportional to an inverse of the precision of the estimates and bar correspond to 95% confidence intervals.

**S9B. Bubble Plots (follow-up period) on Ageusia**

Point sizes are proportional to an inverse of the precision of the estimates.

The regression coefficient was -0.13 (95% confidence interval:-0.37-0.12).(*P*=0.31).

**S9C. Bubble Plots (age) on Ageusia**

Point sizes are proportional to an inverse of the precision of the estimates.

The regression coefficient was -0.06 (95% confidence interval:-0.13-0.01).(*P*=0.12).

**S9D. Bubble Plots (sex) on Ageusia**

Point sizes are proportional to an inverse of the precision of the estimates.

The regression coefficient was -0.09 (95% confidence interval:-0.03-0.02).(*P*=0.49).

**S9E. Funnel Plot of studies reporting on Ageusia**

Egger’s *P* was 0.02, indicating the absence of publication bias.

**S10A. Forest Plot on Alopecia**

Point sizes are proportional to an inverse of the precision of the estimates and bar correspond to 95% confidence intervals.

**S10B. Bubble Plots (follow-up period) on Alopecia**

Point sizes are proportional to an inverse of the precision of the estimates.

The regression coefficient was 0.26 (95% confidence interval:-0.29-0.80).(*P*=0.35).

**S10C. Bubble Plots (age) on Alopecia**

Point sizes are proportional to an inverse of the precision of the estimates.

The regression coefficient was -0.005 (95% confidence interval:-0.05-0.06).(*P*=0.85).

**S10D. Bubble Plots (sex) on Alopecia**

Point sizes are proportional to an inverse of the precision of the estimates.

The regression coefficient was -0.08 (95% confidence interval:-0.16-0.006).(*P*=0.07).

**S10E. Funnel Plot of studies reporting on Alopecia**

Egger’s *P* was 0.03, indicating the presence of publication bias.

**S11A. Forest Plot on Anorexia**

Point sizes are proportional to an inverse of the precision of the estimates and bar correspond to 95% confidence intervals.

**S11B. Bubble Plots (follow-up period) on Anorexia**

Point sizes are proportional to an inverse of the precision of the estimates.

The regression coefficient was 0.34 (95% confidence interval:-0.23-0.90).(*P*=0.24).

**S11C. Bubble Plots (age) on Anorexia**

Point sizes are proportional to an inverse of the precision of the estimates.

The regression coefficient was -0.023 (95% confidence interval:-0.14-0.09).(*P*=0.68).

**S11D. Bubble Plots (sex) on Anorexia**

Point sizes are proportional to an inverse of the precision of the estimates.

The regression coefficient was 0.0013 (95% confidence interval:-0.08-0.08).(*P*=0.97).

**S11E. Funnel Plot of studies reporting on Anorexia**

Egger’s *P* was 0.036, indicating the presence of publication bias.

**S12A. Forest Plot on Anosmia**

Point sizes are proportional to an inverse of the precision of the estimates and bar correspond to 95% confidence intervals.

**S12B. Bubble Plots (follow-up period) on Anosmia**

Point sizes are proportional to an inverse of the precision of the estimates.

The regression coefficient was -0.06 (95% confidence interval:-0.34-0.21).(*P*=0.66).

**S12C. Bubble Plots (age) on Anosmia**

Point sizes are proportional to an inverse of the precision of the estimates.

The regression coefficient was -0.06 (95% confidence interval:-0.11—0.02).(*P*=0.004).

**S12D. Bubble Plots (sex) on Anosmia**

Point sizes are proportional to an inverse of the precision of the estimates.

The regression coefficient was -0.018 (95% confidence interval:-0.05-0.01).(*P*=0.27).

**S12E. Funnel Plot of studies reporting on Anosmia**

Egger’s *P* was 0.006, indicating the presence of publication bias.

**S13A. Forest Plot on Anxiety**

Point sizes are proportional to an inverse of the precision of the estimates and bar correspond to 95% confidence intervals.

**S13B. Bubble Plots (follow-up period) on Anxiety**

Point sizes are proportional to an inverse of the precision of the estimates.

The regression coefficient was 0.16 (95% confidence interval:-0.17-0.50).(*P*=0.34).

**S13C. Bubble Plots (age) on Anxiety**

Point sizes are proportional to an inverse of the precision of the estimates.

The regression coefficient was 0.101 (95% confidence interval:-0.06--.26).(*P*=0.21).

**S13D. Bubble Plots (sex) on Anxiety**

Point sizes are proportional to an inverse of the precision of the estimates.

The regression coefficient was 0.004 (95% confidence interval:-0.007-0.009).(*P*=0.09).

**S13E. Funnel Plot of studies reporting on Anxiety**

Egger’s *P* was 0.004, indicating the presence of publication bias.

**S14A. Forest Plot on Chills**

Point sizes are proportional to an inverse of the precision of the estimates and bar correspond to 95% confidence intervals.

**S14B. Bubble Plots (follow-up period) on Chills**

Point sizes are proportional to an inverse of the precision of the estimates.

The regression coefficient was 0.54 (95% confidence interval:0.07-1.00).(*P*=0.02).

**S14C. Bubble Plots (age) on Chills**

Point sizes are proportional to an inverse of the precision of the estimates.

The regression coefficient was -0.05 (95% confidence interval:-0.33-0.23).(*P*=0.73).

**S14D. Bubble Plots (sex) on Chills**

Point sizes are proportional to an inverse of the precision of the estimates.

The regression coefficient was 0.04 (95% confidence interval:-0.01-0.1).(*P*=0.13).

**S14E. Funnel Plot of studies reporting on Chills**

Egger’s *P* was 0.005, indicating the presence of publication bias.

**S15A. Forest Plot on Confusion**

Point sizes are proportional to an inverse of the precision of the estimates.

**S15B. Bubble Plots (follow-up period) on Confusion**

Point sizes are proportional to an inverse of the precision of the estimates.

The regression coefficient was 0.01 (95% confidence interval:-0.27-0.29).(*P*=0.94).

**S15C. Bubble Plots (age) on Confusion**

Point sizes are proportional to an inverse of the precision of the estimates.

The regression coefficient was -0.10 (95% confidence interval:-0.21-0.006).(*P*=0.05).

**S15D. Bubble Plots (sex) on Confusion**

Point sizes are proportional to an inverse of the precision of the estimates.

The regression coefficient was 0.02 (95% confidence interval:-0.03-0.06).(*P*=0.44).

**S15E. Funnel Plot of studies reporting on Confusion**

Egger’s *P* was 0.12, indicating the presence of publication bias.

**S16A. Forest Plot on Cough**

Point sizes are proportional to an inverse of the precision of the estimates.

**S16B. Bubble Plots (follow-up period) on Cough**

Point sizes are proportional to an inverse of the precision of the estimates.

The regression coefficient was -0.025 (95% confidence interval:-0.29-0.24).(*P*=0.85).

**S16C. Bubble Plots (age) on Cough**

Point sizes are proportional to an inverse of the precision of the estimates.

The regression coefficient was -0.028 (95% confidence interval:-0.06-0.01).(*P*=0.13).

**S16D. Bubble Plots (sex) on Cough**

Point sizes are proportional to an inverse of the precision of the estimates.

The regression coefficient was -0.03 (95% confidence interval:-0.06—0.006).(*P*=0.01).

**S16E. Funnel Plot of studies reporting on Cough**

Egger’s *P* was 0.02, indicating the presence of publication bias.

**S17A. Forest Plot on Depression**

Point sizes are proportional to an inverse of the precision of the estimates.

**S17B. Bubble Plots (follow-up period) on Depression**

Point sizes are proportional to an inverse of the precision of the estimates.

The regression coefficient was 0.26 (95% confidence interval:0.05-0.47).(*P*=0.015).

**S17C. Bubble Plots (age) on Depression**

Point sizes are proportional to an inverse of the precision of the estimates.

The regression coefficient was 0.11 (95% confidence interval:0.02-0.19).(*P*=0.02).

**S17D. Bubble Plots (sex) on Depression**

Point sizes are proportional to an inverse of the precision of the estimates.

The regression coefficient was 0.013 (95% confidence interval:-0.03-0.05).(*P*=0.53).

**S17E. Funnel Plot of studies reporting on Depression**

Egger’s *P* was 0.01, indicating the presence of publication bias.

**S18A. Forest Plot on Diarrhea**

Point sizes are proportional to an inverse of the precision of the estimates.

**S18B. Bubble Plots (follow-up period) on Diarrhea**

Point sizes are proportional to an inverse of the precision of the estimates.

The regression coefficient was 0.28 (95% confidence interval:-0.09-0.64).(*P*=0.13).

**S18C. Bubble Plots (age) on Diarrhea**

Point sizes are proportional to an inverse of the precision of the estimates.

The regression coefficient was -0.01 (95% confidence interval:-0.11-0.09).(*P*=0.80).

**S18D. Bubble Plots (sex) on Diarrhea**

Point sizes are proportional to an inverse of the precision of the estimates.

The regression coefficient was -0.002 (95% confidence interval:-0.04-0.04).(*P*=0.92).

**S18E. Funnel Plot of studies reporting on Diarrhea**

Egger’s *P* was 0.01, indicating the presence of publication bias.

**S19A. Forest Plot on Dyspnea**

Point sizes are proportional to an inverse of the precision of the estimates.

**S19B. Bubble Plots (follow-up period) on Dyspnea**

Point sizes are proportional to an inverse of the precision of the estimates.

The regression coefficient was 0.47 (95% confidence interval:0.18-0.76).(*P*=0.002).

**S19C. Bubble Plots (age) on Dyspnea**

Point sizes are proportional to an inverse of the precision of the estimates.

The regression coefficient was 0.04 (95% confidence interval:-0.04-0.12).(*P*=0.30).

**S19D. Bubble Plots (sex) on Dyspnea**

Point sizes are proportional to an inverse of the precision of the estimates.

The regression coefficient was -0.003 (95% confidence interval:-0.04-0.03).(*P*=0.86).

**S19E. Funnel Plot of studies reporting on Dyspnea**

Egger’s *P* was 0.09, indicating the presence of publication bias.

**S20A. Forest Plot on Fatigue**

Point sizes are proportional to an inverse of the precision of the estimates.

**S20B. Bubble Plots (follow-up period) on Fatigue**

Point sizes are proportional to an inverse of the precision of the estimates.

The regression coefficient was 0.45 (95% confidence interval:0.12-0.78).(*P*=0.007).

**S20C. Bubble Plots (age) on Fatigue**

Point sizes are proportional to an inverse of the precision of the estimates.

The regression coefficient was 0.45 (95% confidence interval:-0.06-0.06).(*P*=0.98).

**S20D. Bubble Plots (sex) on Fatigue**

Point sizes are proportional to an inverse of the precision of the estimates.

The regression coefficient was 0.004 (95% confidence interval:-0.02-0.04).(*P*=0.82).

**S20E. Funnel Plot of studies reporting on Fatigue**

Egger’s *P* was 0.004, indicating the presence of publication bias.

**S21A. Forest Plot on Fever**

Point sizes are proportional to an inverse of the precision of the estimates.

**S21B. Bubble Plots (follow-up period) on Fever**

Point sizes are proportional to an inverse of the precision of the estimates.

The regression coefficient was -0.04 (95% confidence interval:-0.36-0.27).(*P*=0.79).

**S21C. Bubble Plots (age) on Fever**

Point sizes are proportional to an inverse of the precision of the estimates.

The regression coefficient was -0.18 (95% confidence interval:-0.28—0.08).(*P*=0.0002).

**S21D. Bubble Plots (sex) on Fever**

Point sizes are proportional to an inverse of the precision of the estimates.

The regression coefficient was -0.06 (95% confidence interval:-0.09—0.02).(*P*=0.001).

**S21E. Funnel Plot of studies reporting on Fever**

Egger’s *P* was 0.13, indicating the absence of publication bias.

**S22A. Forest Plot on Insomnia**

Point sizes are proportional to an inverse of the precision of the estimates.

**S22B. Bubble Plots (follow-up period) on Insomnia**

Point sizes are proportional to an inverse of the precision of the estimates.

The regression coefficient was 0.51 (95% confidence interval:0.06-0.96).(*P*=0.03).

**S22C. Bubble Plots (age) on Insomnia**

Point sizes are proportional to an inverse of the precision of the estimates.

The regression coefficient was -0.05 (95% confidence interval:-0.25-0.16).(*P*=0.66).

**S22D. Bubble Plots (sex) on Insomnia**

Point sizes are proportional to an inverse of the precision of the estimates.

The regression coefficient was 0.05 (95% confidence interval:0.01-0.09).(*P*=0.008).

**S22E. Funnel Plot of studies reporting on Insomnia**

Egger’s *P* was 0.15, indicating the absence of publication bias.

**S23A. Forest Plot on Memory impairment**

Point sizes are proportional to an inverse of the precision of the estimates.

**S23B. Bubble Plots (follow-up period) on Memory impairment**

Point sizes are proportional to an inverse of the precision of the estimates.

The regression coefficient was 0.42 (95% confidence interval:0.10-0.74).(*P*=0.01).

**S23C. Bubble Plots (age) on Memory impairment**

Point sizes are proportional to an inverse of the precision of the estimates.

The regression coefficient was 0.02 (95% confidence interval:-0.09-0.13) (*P*=0.70)

**S23D. Bubble Plots (sex) on Memory impairment**

Point sizes are proportional to an inverse of the precision of the estimates.

The regression coefficient was 0.04 (95% confidence interval:-0.01-0.09) (*P*=0.13)

**S23E. Funnel Plot of studies reporting on Memory impairment**

Egger’s *P* was <0.001, indicating presence of publication bias.

**S24A. Forest Plot on Nasal blockage**

Point sizes are proportional to an inverse of the precision of the estimates.

**S24B. Bubble Plots (follow-up period) on Nasal blockage**

Point sizes are proportional to an inverse of the precision of the estimates.

The regression coefficient was -0.38 (95% confidence interval:-0.73—0.04).(*P*=0.03).

**S24C. Bubble Plots (age) on Nasal blockage**

Point sizes are proportional to an inverse of the precision of the estimates.

The regression coefficient was 0.03 (95% confidence interval:-0.06-0.12).(*P*=0.52).

**S24D. Bubble Plots (sex) on Nasal blockage**

Point sizes are proportional to an inverse of the precision of the estimates.

The regression coefficient was -0.07 (95% confidence interval:-0.13—0.02).(*P*=0.0098).

**S24E. Funnel Plot of studies reporting on Nasal blockage**

Egger’s *P* was 0.21, indicating the absence of publication bias.

**S25A. Forest Plot on Nausea**

Point sizes are proportional to an inverse of the precision of the estimates.

**S25B. Bubble Plots (follow-up period) on Nausea**

Point sizes are proportional to an inverse of the precision of the estimates.

The regression coefficient was 0.54 (95% confidence interval:0.19-0.89).(*P*=0.003).

**S25C. Bubble Plots (age) on Nausea**

Point sizes are proportional to an inverse of the precision of the estimates.

The regression coefficient was -0.08 (95% confidence interval:-0.24-0.08).(*P*=0.31).

**S25D. Bubble Plots (sex) on Nausea**

Point sizes are proportional to an inverse of the precision of the estimates.

The regression coefficient was 0.01 (95% confidence interval:-0.05-0.08).(*P*=0.68).

**S25E. Funnel Plot of studies reporting on Nausea**

Egger’s *P* was 0.018, indicating the presence of publication bias.

**S26A. Forest Plot on Palpitation**

Point sizes are proportional to an inverse of the precision of the estimates.

**S26B. Bubble Plots (follow-up period) on Palpitation**

Point sizes are proportional to an inverse of the precision of the estimates.

The regression coefficient was 0.30 (95% confidence interval:-0.29-0.89).(*P*=0.32).

**S26C. Bubble Plots (age) on Palpitation**

Point sizes are proportional to an inverse of the precision of the estimates.

The regression coefficient was 0.08 (95% confidence interval:-0.02-0.28).(*P*=0.44).

**S26D. Bubble Plots (sex) on Palpitation**

Point sizes are proportional to an inverse of the precision of the estimates.

The regression coefficient was -0.007 (95% confidence interval:-0.06-0.08).(*P*=0.84).

**S26E. Funnel Plot of studies reporting on Palpitation**

Egger’s *P* was 0.15, indicating the presence of publication bias.

**S27A. Forest Plot on Rhinorrhea**

Point sizes are proportional to an inverse of the precision of the estimates.

**S27B. Bubble Plots (follow-up period) on Rhinorrhea**

Point sizes are proportional to an inverse of the precision of the estimates.

The regression coefficient was 0.10 (95% confidence interval:-0.27-0.47).(*P*=0.59).

**S27C. Bubble Plots (age) on Rhinorrhea**

Point sizes are proportional to an inverse of the precision of the estimates.

The regression coefficient was 0.05 (95% confidence interval:-0.1-0.19).(*P*=0.50).

**S27D. Bubble Plots (sex) on Rhinorrhea**

Point sizes are proportional to an inverse of the precision of the estimates.

The regression coefficient was -0.007 (95% confidence interval:-0.06-0.05).(*P*=0.80).

**S27E. Funnel Plot of studies reporting on Rhinorrhea**

Egger’s *P* was 0.02, indicating the presence of publication bias.

**S28A. Forest Plot on Sneezing**

Point sizes are proportional to an inverse of the precision of the estimates.

**S28B. Bubble Plots (follow-up period) on Sneezing**

Point sizes are proportional to an inverse of the precision of the estimates.

The regression coefficient was 0.58 (95% confidence interval:-0.31-1.46).(*P*=0.20).

**S28C. Bubble Plots (age) on Sneezing**

Point sizes are proportional to an inverse of the precision of the estimates.

The regression coefficient was -0.40 (95% confidence interval:-1.03-0.24).(*P*=0.22).

**S28D. Bubble Plots (sex) on Sneezing**

Point sizes are proportional to an inverse of the precision of the estimates.

The regression coefficient was -0.02 (95% confidence interval:-0.13-0.09).(*P*=0.76).

**S28E. Funnel Plot of studies reporting on Sneezing**

Egger’s *P* was 0.56, indicating the absence of publication bias.

**S29A. Forest Plot on Sputum**

Point sizes are proportional to an inverse of the precision of the estimates.

**S29B. Bubble Plots (follow-up period) on Sputum**

Point sizes are proportional to an inverse of the precision of the estimates.

The regression coefficient was -0.22 (95% confidence interval:-0.46-0.01).(*P*=0.07).

**S29C. Bubble Plots (age) on Sputum**

Point sizes are proportional to an inverse of the precision of the estimates.

The regression coefficient was 0.01 (95% confidence interval:-0.07-0.10).(*P*=0.78).

**S29D. Bubble Plots (sex) on Sputum**

Point sizes are proportional to an inverse of the precision of the estimates.

The regression coefficient was 0.01 (95% confidence interval:-0.02-0.04).(*P*=0.49).

**S29E. Funnel Plot of studies reporting on Sputum**

Egger’s *P* was 0.63, indicating the absence of publication bias.

**S30A. Forest Plot on Vertigo (Dizziness)**

Point sizes are proportional to an inverse of the precision of the estimates.

**S30B. Bubble Plots (follow-up period) on Vertigo (Dizziness)**

Point sizes are proportional to an inverse of the precision of the estimates.

The regression coefficient was 0.35 (95% confidence interval:-0.17-0.87).(*P*=0.19).

**S30C. Bubble Plots (age) on Vertigo (Dizziness)**

Point sizes are proportional to an inverse of the precision of the estimates.

The regression coefficient was 0.03 (95% confidence interval:-0.13-0.18).(*P*=0.75).

**S30D. Bubble Plots (sex) on Vertigo (Dizziness)**

Point sizes are proportional to an inverse of the precision of the estimates.

The regression coefficient was -0.002 (95% confidence interval:-0.06-0.06).(*P*=0.96).

**S30E. Funnel Plot of studies reporting on Vertigo (Dizziness)**

Egger’s *P* was 0.014, indicating the absence of publication bias.

**S31A. Forest Plot on Vomiting**

Point sizes are proportional to an inverse of the precision of the estimates.

**S31B. Bubble Plots (follow-up period) on Vomiting**

Point sizes are proportional to an inverse of the precision of the estimates.

The regression coefficient was 0.36 (95% confidence interval:-0.15-0.88).(*P*=0.17).

**S31C. Bubble Plots (age) on Vomiting**

Point sizes are proportional to an inverse of the precision of the estimates.

The regression coefficient was -0.04 (95% confidence interval:-0.27-0.19).(*P*=0.72).

**S31D. Bubble Plots (sex) on Vomiting**

Point sizes are proportional to an inverse of the precision of the estimates.

The regression coefficient was 0.04 (95% confidence interval:-0.06-0.15).(*P*=0.42).

**S31E. Funnel Plot of studies reporting on Vomiting**

Egger’s *P* was 0.002, indicating the presence of publication bias.

**S32A. Forest Plot on Weakness**

Point sizes are proportional to an inverse of the precision of the estimates.

**S32B. Bubble Plots (follow-up period) on Weakness**

Point sizes are proportional to an inverse of the precision of the estimates.

The regression coefficient was 0.13 (95% confidence interval:-0.75-1.01).(*P*=0.76).

**S32C. Bubble Plots (age) on Weakness**

Point sizes are proportional to an inverse of the precision of the estimates.

The regression coefficient was -0.50 (95% confidence interval:-0.70—0.29).(*P*<0.001).

**S32D. Bubble Plots (sex) on Weakness**

Point sizes are proportional to an inverse of the precision of the estimates.

The regression coefficient was -0.009 (95% confidence interval:-0.09-0.07).(*P*=0.84).

**S32E. Funnel Plot of studies reporting on Weakness**

Egger’s *P* was 0.46, indicating the absence of publication bias.

**S33A. Forest Plot on Weight loss**

Point sizes are proportional to an inverse of the precision of the estimates.

**S33B. Bubble Plots (follow-up period) on Weight loss**

Point sizes are proportional to an inverse of the precision of the estimates.

The regression coefficient was -0.96 (95% confidence interval:-2.80-0.88).(*P*=0.31).

**S33C. Bubble Plots (age) on Weight loss**

Point sizes are proportional to an inverse of the precision of the estimates.

The regression coefficient was 0.06 (95% confidence interval:-0.23-0.36).(*P*=0.66).

**S33D. Bubble Plots (sex) on Weight loss**

Point sizes are proportional to an inverse of the precision of the estimates.

The regression coefficient was 0.02 (95% confidence interval:-0.01-0.45).(*P*=0.21).

**S33E. Funnel Plot of studies reporting on Weight loss**

Egger’s *P* was 0.13, indicating the absence of publication bias.
