# Supplementary material for: Incidence of long-term post-acute sequelae of SARS-CoV-2 infection related to pain and other symptoms: A systematic review and meta-analysis
Source: PLoS One. 2023 Nov 29;18(11):e0250909. doi: 10.1371/journal.pone.0250909 (PMC10686440; doi:10.1371/journal.pone.0250909)
Supplement: Attachment — Submitted filename: Response to Reviewers.docx [file pone.0250909.s004.docx]

**Answers to the reviewers’ comments.**

The original reviewers’ comments are expressed in “**bold**” type, and our comments are expressed in “plain” type.

**Reviewer #1: I read with interest about this comprehensive systemic review of post-acute COVID-19 symptoms. Generally, the article was well written with adequate meta-analyses. I still had some concerns about this study.**

Thank you for your comments.

First of all, we attempted to update the latest articles. After screening with the same search strategy, we found a skyrocketing number of articles on this theme: More than 2,000 articles from Scopus; around 20,000 from medRxiv, and more than 400 from EMBASE. After we discussed this, we considered it impossible, within this short period of revision time, to screen, extract data, re-analyze, and extensively rewrite the manuscript. Therefore, we decided to include the only studies you have pointed out, without altering the current search period. We kindly ask for your understanding in advance.

**1. At least some important studies were not included in the current study. I suggest authors include these studies and re-analyze your results.
a. Chopra, V., Flanders, S. A. & O’Malley, M. Sixty-day outcomes among patients hospitalized with COVID-19. Ann. Intern. Med. (2020).
b. Garrigues, E. et al. Post-discharge persistent symptoms and health-related quality of life after hospitalization for COVID-19. J. Infect. 81, e4–e6 (2020).**

We have completed re-analyses after including these studies.

**2. The last literature research was performed on January 15, 2021. The post-acute COVID-19 symptoms had been reported more in detail after that time. Since this study is aimed to be a living meta-analysis, is it feasible for authors to include newer publised studies after Jan 2021. At least the following should be considered:
a. Huang L, Yao Q, Gu X, et al. 1-year outcomes in hospital survivors with COVID-19: a longitudinal cohort study. Lancet. 2021; 398(10302):747-758. →Updated results from reference 31.
b. Moreno-Pérez, O. et al. Post-acute COVID-19 syndrome. Incidence and risk factors: a Mediterranean cohort study. J. Infect. (2021)**

We have completed re-analyses after including Moreno-P**é**rez. As you pointed out, the Huang et al. study is certainly an update of reference 31; however, we did not include this study because it was published far beyond the current search period.

Finally, we have included the three articles that you pointed out.

In addition, we excluded the term “Living” from the manuscript title as this is no longer a “living” meta-analysis.

**Reviewer #2:**

**The authors use a living systematic review and meta-analysis to depict the incidence of long-term post-acute sequelae of SARS-CoV-2 infection related to pain and other symptoms. They defined long-term complications as symptoms from which patients suffered for more than one month after onset of the first COVID-19 symptoms or after discharge from hospital.**

Thank you for your time spent in reviewing our manuscript.

**There are several concerns as follows:

Major concern 1: From the results, pain-related symptoms are much less frequently noticed compared to the secondary outcome, symptoms relevant to pain such as fatigue, insomnia, dyspnea, weakness. I am wondering whether the clinical setting could be a source of selection bias. For example, a patient underwent invasive treatment might be highly sedative. The pain would be masked initially, and its related symptoms may flair up in later disease courses.**

We agree with you that pain-related symptoms are much less frequently noticed. We added this comment as a limitation in the limitations paragraph in the Discussion section. Closely related to your concern 3, we could not perform sensitivity analyses between ICU and non-ICU patients in this revision.

**Major concern 2: Theoretically, all symptoms should be thoroughly observed from estimated onset of the disease. It would be better to have a consistent format for checking up each symptom with daily bases. Since the available published data bear witness to a high heterogeneity, I suggest the authors make a summary table to show the common symptoms according to individual report.**

The top three most frequent symptoms were ranked in each study, and we summarized them in an overall ranking. Our results showed that most frequent symptoms summarized were fatigue (n=20), followed by dyspnea (17), cough (13), anosmia (12) and fever (6).

The detailed results are described in the Results section and eTable 3.

**Major concern 3: The authors smartly described “post intensive care syndrome” or “ICU-acquired weakness" in Discussion Section. However, the sensitivity analyses based on ICU and Non-ICU patients are lacking in present draft. If the authors can provide this assessment, it would be also helpful to clarify the Major concern 1.**

To perform a sensitivity analysis between ICU and non-ICU patients, we thoroughly reviewed all of the studies we included. However, we found that only two of the studies summarized ICU and non-ICU patients separately. Probably, most of patients in the rest of the studies did not appear to be transferred to the ICU. Therefore, a sensitivity analysis was difficult to perform.

**Minor concern, Line 216: COVID-19 survivors were multifarious with "an" incidence of 5-17%.**

Thank you. We corrected this.
