# Supplementary material for: Incidence of long-term post-acute sequelae of SARS-CoV-2 infection related to pain and other symptoms: A systematic review and meta-analysis
Source: PLoS One. 2023 Nov 29;18(11):e0250909. doi: 10.1371/journal.pone.0250909 (PMC10686440; doi:10.1371/journal.pone.0250909)
Supplement: Attachment — Submitted filename: Response to Reviewers.docx [file pone.0250909.s005.docx]

**Responses to the reviewers’ comments**

The original reviewers’ comments are expressed in “**bold**” type, and our comments are expressed in “plain” type.

**Reviewer #1: The authors addressed reviewer's comment and revised the manuscript accordingly. I suggest the current study accepted for publication.**

Thank you very much for your comment and recommendation.

**Reviewer #3: The authors submitted a revised version of the article along with thorough explanation of the way by which the changes and corrections were made. I have no serious flaws to the article in its revised version.**

Thank you for your comments.

**Reviewer #4: The authors submitted a revised version of the paper along with the clear explanation if the ways by which the corrections were made. I have no serious flaws to the article in its revised version.**

Thank you for your comments.

**Reviewer #5: This meta analysis is totally out of date. The last search was January 2021, now we are September 2022. One year in COVID-19 research implies that several papers and MA about post-COVID pain have been published. This paper does not add nothing to the literature and does not include most updated data.**

We totally agree with your comments.

To be honest, this paper has a long history. It was originally published in MedRxiv on April 8, 2021, before its submission to *PLOS On*e. The manuscript revision process took more than six months, after which the manuscript was officially accepted by PLOS One on August 13, 2022.
One month later, after our payment to the journal, however, acceptance was suddenly retracted by the editorial office due to a potential concern regarding the quality of the peer review process. So, this explains the delay.

During this time, thousands of articles on long COVID were published, so we think that would be impossible, within this short period of revision time, to screen and extract new data, re-analyze it, and extensively rewrite the manuscript. We believe that the previous reviewers were happy with our response to these circumstances. We kindly ask for your understanding in advance.

However, as the editor requested, we attempted to discuss our results in the context of the recently published literature. We found one similar meta-analysis published by Fernandez-de-las-Penas et al. in 2022 (This paper was submitted on 26 May, 2021, when our preprint has already published; therefore, we were first). In the paper, the most frequent pain-related symptoms and their incidences were comparable with those in our studies. For instance, chest pain is the most reported, and its incidence ranges between 7.8-23.6%. We added this in the Discussion section as follows: “During the course of our study, a similar meta-analysis related with pain were published [63]. Although this might weaken the originality of our study, in the publication, the most frequently reported pain-related symptoms and their incidence were comparable with those reported in our studies. For instance, chest pain is the most reported symptoms, and its incidence ranges between 7.8-23.6%.

**Reviewer #6: This manuscript describes a systematic review and meta-analysis to estimate the incidence rate for pain and other symptoms due to “long COVID.”
This study used several large electronic databases (including MediRxiv and BioRxiv) to search articles before Jan 15, 2021. In total, 1290 articles were identified in the literature search, and finally, 35 studies were included in the study after a rigorous screening and selection process. The analyses followed a PRISMA standard for meta-analysis, and the inter-study heterogeneity was assessed using I^2 statistics. In addition, the authors investigated the potential source of heterogeneity using meta-regression with a mixed-effects model. Furthermore, the publication bias was assessed by Egger’s P statistics and funnel plots.
Overall, the study method is solid and rigorous. This study provides comprehensive results for long COVID related pain and other symptoms.
I have two questions just for curiosity.
1. The result “every one month of follow-up corresponds to an increase of 1.45 units (45% increase) in prevalence in patients who developed neuralgia after acute COVID-19 infection” indicate the longer follow-up time is associated high incidence rate of neuralgia. Is that because neuralgia will take time to develop, so the studies with short follow-up time can not detect the development of neuralgia?**

First of all, we know you are busy, and we appreciate you taking the time to write your comments.

Regarding neuralgia, there are only three studies with high heterogeneity. Because of the small amount of available information, definite conclusions cannot be drawn. This statistic (45% increase per 1.45 units) is just an estimation calculated from the limited sample data available. We have already addressed this previously in the Discussion section as follows: “In any case, we are aware that these statistical models are preliminary and exploratory, and 53% of symptoms were not explainable despite three typical covariates being incorporated into the model. Symptoms of long COVID are reported to be on-and-off, cyclic or multiphasic [5], which is why the linear regression model did not fit well.”

**2. Will different regions of study (e.g. Europe, US and China) be a potential factor contributing to the heterogeneity, as people from different regions may have different tolerances or thresholds of pain?**

We think it’s probably yes. According to the meta-analysis published by Alkodyami et al. in 2022, lower incidences of fatigue, dyspnea, and loss of smell and taste were reported in Asian populations. However, meta-regression analysis with region as the covariate is beyond the aim of our study and was not included in our initial protocol, so we added this as a limitation in the Discussion section as follows: “Lastly, studies performed in different geographical regions might be a potential factor contributing to the heterogeneity, which was suggested in a recent meta-analysis [64].”

.
